# Cyanobacteria in sustainable agriculture: from bioactive metabolites toward mechanistically validated biostimulants

**DOI:** 10.1007/s00203-026-05265-y

**Published:** 2026-10-15

**Authors:** Mauricio Junior Machado, Bruno Bachiega Navarro, Fernanda Rios Jacinavicius, Marli Fátima Fiore

**Affiliations:** 1 Centro de Energia Nuclear na Agricultura (CENA), Universidade de São Paulo (USP), Piracicaba, 13416-000 SP Brazil; 2 Escola Superior de Agricultura “Luiz de Queiroz” (ESALQ), Universidade de São Paulo (USP), Piracicaba, 13416-900 SP Brazil

**Keywords:** Agricultural biostimulants, Bioactive metabolites, Cyanobacteria, Microbial biotechnology, Plant–microbe interactions, Stress mitigation

## Abstract

Cyanobacteria are emerging as multifunctional components of sustainable agroecosystems, with the potential to influence nutrient cycling, soil structure, plant-associated microbiomes, and crop responses to environmental stress. This review integrates current knowledge of the molecular and physiological mechanisms underlying these effects, emphasizing nitrogen fixation, nutrient mobilization, phytohormone biosynthesis, extracellular polymeric substances, biocontrol-related metabolites, and stress mitigation. Despite the growing number of cyanobacterial metabolites and traits associated with plant growth promotion and stress tolerance, direct functional and causal evidence linking specific compounds or pathways to plant responses remains limited. The work therefore critically evaluates the evidence supporting cyanobacterial biostimulant functions, distinguishing associative observations from functional and causal validation, while considering translational evidence from controlled experiments to field conditions. This review examines whole-cell inoculants, metabolite-based formulations, hybrid strategies, synthetic consortia, and engineered cyanobacteria in terms of mechanistic support, formulation, reproducibility, scalability, and agricultural applicability. Biosafety, particularly strain-level cyanotoxin risk and the need for appropriate toxicological screening, is also considered an important component of biostimulant development. Because performance depends on interactions among cyanobacterial strain, host plant, formulation, soil properties, microbiomes, and environmental conditions, future progress will require integrating mechanistic biology, metabolomics, formulation development, safety assessment, and field-scale validation. Bridging mechanistic and translational evidence will be essential to convert cyanobacterial functional diversity into reliable, targeted, and scalable technologies for sustainable agriculture.

## Introduction

Microbial biostimulants are increasingly explored as tools to support crop performance, nutrient use, and stress tolerance while complementing conventional fertilization and reducing reliance on chemical inputs under increasingly challenging environmental conditions (Fagunwa et al., 2020; Castiglione et al. 2021; Akinsemolu 2023; Mannino 2025; Navarro et al. 2025). As sustainable crop production becomes an increasingly urgent global priority, next-generation biostimulants seek greater efficacy, formulation stability, and environmental compatibility through improved biological sources and delivery strategies (Richa 2024).

Among the diverse microorganisms investigated as agricultural biostimulants, cyanobacteria are particularly promising because of their ecological diversity, tolerance to nutrient limitation, desiccation, radiation, and oxidative stress, and capacity to produce a broad repertoire of bioactive metabolites (Patzelt et al. 2014; Pushkareva et al. 2015; Gaysina et al. 2019; Jacinavicius et al. 2021; Waditee-Sirisattha and Kageyama 2022). These include pigments, polysaccharides, phytohormone-like compounds, antioxidants, siderophores, peptides, and terpenoids that may influence plant physiology, nutrient dynamics, and soil processes (Kumar et al. 2019; Jones et al. 2021; Machado et al. 2025; Weiss et al. 2025a). This functional diversity has positioned cyanobacteria as promising biological platforms for agricultural biostimulants (Jurado-Flores et al. 2025).

Research has progressed from studies of natural nitrogen-fixing associations, particularly the *Azolla*–cyanobiont system in rice-based agriculture (Raja et al. 2012; Yadav et al. 2014), toward mechanistic and applied investigations supported by genomics, metabolomics, analytical chemistry, and synthetic biology. Current evidence links cyanobacteria to nitrogen fixation, phosphate solubilization, extracellular polymeric substance production, rhizosphere interactions, and modulation of stress-responsive pathways (Belnap & Lange, 2003; Ahmed et al. 2010; Prasanna et al. 2010; Joshi et al. 2020; Sun et al. 2026).

Despite this progress, translating promising experimental results into consistent field performance remains a major challenge. Cyanobacterial effects vary substantially among strains, formulations, crops, environmental conditions, and application strategies, while the molecular basis of many reported plant responses remains insufficiently resolved. In particular, detecting a metabolite alongside a plant response is often interpreted as evidence of biological function, although direct validation through compound isolation, dose–response assays, or functional perturbation remains limited. This gap makes it difficult to identify robust strains and metabolites and translate them into reproducible agricultural applications.

Recent reviews have addressed cyanobacteria-based biofertilizers in arid and semi-arid agroecosystems (Bibi et al. 2024), broadly synthesized their roles in plant growth promotion, stress alleviation, and phytoremediation (Nawaz et al. 2024), integrated microalgae and cyanobacteria within the wider context of biofertilizers, biostimulants, biopesticides, and circular agricultural systems (Sun et al. 2026), and discussed commercially available cyanobacteria-based products and associated economic and regulatory constraints (Santini et al. 2021; Righini et al. 2022). However, comparatively less attention has been given to critically assessing the strength of experimental evidence linking specific cyanobacterial traits and metabolites to plant responses. Therefore, rather than cataloging reported agricultural benefits, this review critically evaluates evidence for cyanobacterial mechanisms and bioactive metabolites, distinguishes associative observations from functional and causal validation, and examines how these evidence gaps affect the development of reproducible, field-ready cyanobacterial biostimulants.

As a narrative review, the literature was identified through targeted searches using combinations of terms related to cyanobacteria, agriculture, biostimulants, biofertilizers, plant growth, stress responses, bioactive metabolites, and the specific mechanisms addressed in each section, complemented by screening references to relevant studies. Particular attention was given to studies providing experimental evidence linking cyanobacterial strains, preparations, or metabolites to plant, pathogen, or soil responses.

## Ecological and biological importance of cyanobacterial interactions

### Cyanobacteria–plant associations

Cyanobacteria form associations with diverse hosts, including algae, fungi, animals, and plants (Cameron et al. 2024; Machado et al. 2026). In plants, cyanobionts occur in bryophytes, *Azolla*, cycads, and *Gunnera*, and facultative associations with nitrogen-fixing cyanobacteria have also been reported in Amaranthaceae, Fabaceae, and Poaceae (Rai et al. 2000; Delaux et al. 2015; Devaprakash et al. 2024). These interactions commonly involve reciprocal nutrient exchange, in which diazotrophic cyanobacteria provide reduced nitrogen while host-derived carbon supports cyanobiont metabolism (Xu and Wang 2023). Nitrogen-fixing cyanobacteria from the order Nostocales, particularly *Nostoc* and related genera such as *Anabaena*, *Trichormus*, and *Calothrix*, occupy specialized host structures, including thallus and leaf cavities, coralloid roots, and stem glands (Dodds et al. 1995; Bergman 2005; Adams et al. 2013).

Among these associations, the *Azolla*–cyanobiont system is a major ecological and agricultural model of biological nitrogen input in rice-based systems (Yadav et al. 2014). Within *Azolla* leaf cavities, the cyanobiont fixes atmospheric N₂ and transfers reduced nitrogen to the fern, supporting rapid nitrogen accumulation and subsequent soil enrichment when the biomass is incorporated as green manure. This functional integration underpins the use of *Azolla* in paddy biofertilization and other applications, including animal feed and heavy-metal remediation, although some *Azolla* species can become invasive outside managed systems (Raja et al. 2012; Sood et al. 2012; Mahanty et al. 2017; Pinero-Rodríguez et al. 2021). In agricultural contexts, this association may therefore reduce dependence on mineral nitrogen inputs (Yao et al. 2018).

The *Azolla* cyanobiont provides a mechanistic model of host-associated specialization. The genome of ‘*Nostoc azollae*’ 0708 shows extensive pseudogenization and accumulation of insertion sequences, with losses affecting DNA replication and repair, nutrient uptake, and central metabolism, whereas functions supporting cell differentiation and nitrogen fixation remain conserved (Ran et al. 2010; Vigil-Stenman et al. 2015). Proteomic comparison with the related free-living strain *Nostoc* sp. PCC 73102 revealed enrichment of the nitrogenase components NifH and NifK, together with proteins involved in folding, assembly, degradation, and oxidative-stress protection, consistent with a symbiotic metabolism oriented toward nitrogen fixation (Ekman et al. 2008). Together, genomic and proteomic evidence shows how genome reduction and metabolic specialization help maintain the *Azolla*–cyanobiont symbiosis (Adams et al. 2013).

Symbiotically competent *Nostoc* strains provide useful models for understanding the early processes involved in cyanobacterial recognition and colonization of plants. Plant-derived hormogonia-inducing factors trigger the differentiation of motile hormogonia, which respond to plant-derived signals, attach to roots, and initiate colonization (Nilsson et al. 2002; Adams and Duggan 2011). These signals include flavonoids and diacylglycerols associated with hormogonium differentiation, while soluble sugars (arabinose, galactose, and glucose) can act as chemoattractants for motile hormogonia (Nilsson et al. 2006; Hashidoko et al. 2019). Following successful colonization, cyanobacteria can differentiate heterocysts within root tissues and exhibit nitrogenase activity, which may promote plant growth under nitrogen-limiting conditions (Nilsson et al. 2005; Álvarez et al. 2020).

Importantly, the chemotactic responses are not restricted to natural cyanobacterial hosts. *Nostoc* strains 8964:3 and PCC 73102 responded to extracts of both natural hosts and nonhost plants, including *Oryza sativa*, suggesting that chemo-attracting compounds may be widespread among plants (Nilsson et al. 2006). However, the specific plant-derived compound(s) responsible for the attraction elicited by these complex plant extracts remain unidentified. Proteomic analyses and reduced colonization of rice mutants impaired in the common symbiosis signaling pathway further indicate reciprocal molecular responses and the involvement of conserved host signaling components, including POLLUX, which participates in Ca²⁺ spiking; the calcium/calmodulin-dependent protein kinase CCaMK, which decodes Ca²⁺ oscillations; and the downstream transcriptional regulator CYCLOPS (Álvarez et al. 2022). However, colonization can depend on strain–host compatibility, tissue accessibility, signaling, and environmental conditions, limiting the direct translation of model associations to different crops (Issa et al. 2014; Cameron et al. 2024). Chemotactic responses themselves are also environmentally sensitive, with elevated temperature and darkness reducing hormogonial attraction in experimental assays (Nilsson et al. 2006).

Beyond specialized symbioses, cyanobacteria can persist in crop rhizospheres and colonize root and leaf surfaces, where they may contribute to nutrient exchange and interact with resident microbial communities (Prasanna et al. 2010). Non-heterocystous genera, including *Synechocystis*, *Chroococcidiopsis*, *Leptolyngbya*, and *Phormidium*, have also been associated with wheat, mung bean, and pea (Ahmed et al. 2010; Prasanna et al. 2013a). However, the mechanisms governing persistence, compatibility, and functional integration remain poorly resolved and strongly context-dependent. Artificial associations, co-cultures, and formulated consortia therefore provide tractable systems for identifying signals and traits that support reproducible plant and soil responses (Kollmen and Strieth 2022; Solomon et al. 2023). Together, natural and engineered associations may bridge ecological observations and the development of more predictable cyanobacteria-based bioinoculants.

### Cyanobacteria in microbial communities and soil ecosystems

Cyanobacteria are primary producers in many microbial assemblages, where fixed carbon and released metabolites support heterotrophic growth and nutrient recycling (Kulasooriya 2012; Christie-Oleza et al. 2017; Devaprakash et al. 2024). Their nutrient-responsive exometabolome encompasses free amino acids, including glutamate, proline, valine, methionine, tryptophan, and arginine, as well as dipeptides such as Gly-Ile, Phe-Ile, Val-Arg, and Leu-Pro, whose extracellular abundance varies with nutrient availability and physiological state (Jain et al. 2025; Peck et al. 2025). These released metabolites may support metabolic exchanges with associated microorganisms and, in turn, influence microbial interactions within cyanobacteria-associated communities. Within the cyanosphere, these exchanges may support bacterial colonization, ammonification, and nitrogen retention within microbial loops (Yan et al. 2023). Studies of freshwater and terrestrial cyanobacterial communities have identified associated microbiomes with genomic potential for complementary functions related to nutrient acquisition and cycling, metabolite exchange, and polysaccharide utilization (Kust et al. 2025; Palmer et al. 2026). These systems provide tractable models for investigating metabolic complementarity between cyanobacteria and their associated microorganisms. However, the specific interactions identified in these communities should not be assumed to operate the same way in agricultural soils, where plant-derived inputs, resident microbial communities, management practices, and distinct physicochemical and spatial conditions may reshape microbial interactions.

In terrestrial and agricultural soils, cyanobacterial biomass supplies fixed carbon, whereas diazotrophic strains can contribute fixed nitrogen. Cyanobacteria also release organic acids, polysaccharides, and other extracellular products associated with microbial activity, aggregation, and nutrient retention (GR et al., 2021; Iniesta-Pallarés et al. 2023). Heterotrophic partners can mineralize cyanobacterial biomass and exudates, returning nutrients to soil–plant systems (Devaprakash et al. 2024). Across paddy soils, cyanobacterial inoculation increased cyanobacterial and *nifH* gene abundance, leading to soil-specific changes in carbon- and nitrogen-cycling markers and rhizosphere colonization (Kour et al. 2024). Partial replacement of mineral nitrogen with nitrogen-fixing cyanobacteria in a field Mollisol also shifted bacterial community composition and increased microbial carbon utilization (Liang et al. 2024).

In degraded soils characterized by poor aggregate stability, low organic matter, low microbial activity, low water-holding capacity, and susceptibility to surface erosion (Macedo et al. 2023), cyanobacterial filaments and extracellular polymers may bind particles and stabilize surfaces, creating more favorable microsites for microbial establishment. Inoculation with *Leptolyngbya* sp. and *Scytonema* sp. promoted surface gypsum formation and soil stability without detectable disruption of native bacterial communities in the tested system (Jiménez-González et al. 2022). Similarly, combining a native cyanobacterial consortium with paper-waste amendments improved aggregation, water retention, soil organic carbon, and nitrogen in dryland mesocosm and field experiments, highlighting the importance of formulation and substrate context (Maggioli et al. 2025). Because cyanobacteria-mediated interactions are shaped by root exudates, resident microbiota, soil physicochemical properties, and environmental stress (Philippot et al. 2024), inoculation may alter microbial community composition, diversity, or the relative abundance of particular taxa. However, such taxonomic shifts alone do not establish functional improvement: enrichment or depletion of specific microbial groups does not necessarily imply enhanced nutrient cycling, greater nutrient availability, improved plant performance, or increased community resistance and recovery following disturbance. Linking metacommunity restructuring to these outcomes therefore requires complementary functional measurements, such as nutrient transformation rates, soil physicochemical responses, plant performance, and microbial resistance or recovery after stress (Priya et al. 2015; Pound et al. 2021). Identifying reproducible metabolic exchanges and compatible microbial partners is therefore central to designing inoculants and consortia that remain functional across changing soils and environments.

## Mechanisms underlying the effects of cyanobacteria-based biostimulants

Whole-cell inoculants, symbiotic associations, extracts, and hydrolysates can influence crop and soil performance through interacting nutritional, structural, signaling, and protective mechanisms. These include N₂ fixation, nutrient mobilization, extracellular polymeric substance production, phytohormone-related activity, biocontrol, and stress mitigation (Table 1).

**Table 1:** Overview of agricultural functions of cyanobacteria

Applications	Mechanisms	Metabolites	Cyanobacteria	References
Fertilizer				
Nitrogen	N2 fixation	Ammonia and nitrogen-derived compounds	*Anabaena* sp., *Aulosira* sp., *Calothrix* sp., *Cylindrospermum* sp., *Nostoc* sp., *Tolypothrix* sp., *Scytonema* sp.	Álvarez et al. (2020); Bidyarani et al. (2016); Jiménez-Ríos et al. (2024); Prasanna et al. 2013b), 2014), (2017);
Phosphorus and Potassium	Nutrient solubilization and uptake	Organic acids, phosphatases, and EPS	*Anabaena sp.*,* Aphanothece* sp., *Nostoc* sp., *Westiellopsis prolifica*,	Afkairin et al. (2021); Nawaz et al. (2024); Toribio et al. (2020); Mutale-Joan et al. (2020); Yandigeri et al. (2011)
Iron deficiency	Iron chelates	Siderophores	*Anabaena* sp., *Nodularia* sp., *Nostoc* sp., *Synechococcus* sp., *Trichormus* sp.	Brick et al. (2025); Roque et al. (2023); Toribio et al. (2020)
Organic matter	Biomass	Organic carbon and nutrients	*Anabaena* sp., *Aulosira* sp., *Cylindrospermum* sp., *Nostoc* sp., *Tolypothrix* sp., *Scytonema* sp.	Do Nascimento et al. (2019). Maqubela et al. (2009)
Soil Properties				
Chemical, Moisture, Aggregates, and Texture	Soil conditioning	EPS Production	*Leptolyngbya* sp., *Microcoleus* sp., *Nostoc* sp., *Oscillatoria* sp., *Phormidium* sp., *Scytonema* sp.	Adessi et al. (2018); Asghari et al. (2022); Chamizo et al. (2018), (2020); Chua et al. (2020); Do Nascimento et al. (2019); Malam Issa et al. ( 2007); Maqubela et al. (2009); Nisha et al. (2018)
Soil biology	Microbiome functioning	Enzymes, nutrients, vitamins, and others	*Calothrix elenkinii*,* Nodosilinea* sp., *Phormidium* sp.	Iniesta-Pallarés et al. (2023); Nisha et al. (2018); Prasanna et al. (2016); Priya et al. (2015);
Plant growth promotion				
Biostimulant	Phytohormones	Auxins, cytokinins, gibberellins, and vitamins	*Arthrospira sp.*,* Anabaena sp.*,* Calothrix* sp., *Chroococcidiopsis* sp., *Leptolyngbya sp.*,* Microcoleus sp.*,* Nodosilinea sp.*,* Nostoc* sp., *Oscillatoria* sp., *Phormidium* sp., *Plectonema* sp., *Scytonema* sp., *Trichocoleus* sp.	Chua et al. (2020); Hashtroudi et al. (2013); Hussain and Hasnain (2011); Hussain et al. (2015); Mógor et al. (2018a b); Osman et al. (2010); Roque et al. (2023); Singh et al. (2011); Toribio et al. (2020); Wenz et al. (2019); Zhou et al. (2020)
Plant protection				
Bacteria, fungi, and nematode control	Antibiotic and toxin compounds	Peptides, alkaloids, terpenes	*Anabaena* sp., *Calothrix sp.*, *Nostoc *sp., *Nodularia* sp. *Oscillatoria* sp.,	Chaudhary et al. (2012); Hamouda and El-Ansary (2017); Khan et al. (2007); Prasanna et al. 2013a), 2016); Zhou et al. (2020)
Plant abiotic stress mitigation				
Drought and salinity	Osmoregulation, Antioxidant defense	EPS, amino acids, Pigments, enzymes, Glutathione	*Anabaena sp.*,* Leptolyngbya* sp., *Limnospira* sp., *Microcoleus* sp., *Nostoc* sp., *Neowestiellopsis* sp., *Oscillatoria* sp.	Abbasi and Mohammadi (2023); Cuddy et al. (2013);Do Nascimento et al. (2019); Gharemahmudli and Sadeghi (2025); Gr et al. (2021); Nisha et al. (2018); Sneha et al. (2024)
Bioremediation				
Herbicides and Insecticides	Metabolization, Biosorption	Enzymes, Glutathione	*Anabaena* sp., *Lyngbya* sp., *Microcystis* sp., *Nostoc* sp., *Limnospira sp.*,* Scytonema sp. Fischerella sp.*,* Synechocystis sp.*,* Phormidium sp.*	Aminfarzaneh & Duygu (2010); Forlani et al. (2008); Gaafar et al. (2022); Tiwari et al. (2017)
Heavy metal	Bioaccumulation, Biosorption, Immobilization	EPS Production	*Anabaena* sp., *Cyanothece* sp., *Leptolyngbya* sp., *Microcystis* sp., *Nostoc* sp., *Synechococcus* sp.	Al-Amin et al. (2021); Mao et al. (2023); Pagli et al. (2024); Seifikalhor et al. (2020)

### Nitrogen fixation and nutrient acquisition

Cyanobacteria contribute to nutrient acquisition through atmospheric N₂ fixation, phosphorus solubilization, and changes in nutrient availability and uptake (Bibi et al. 2024). Cyanobacteria-based biofertilizers have also been associated with lower mineral-fertilizer requirements and production costs in specific crop systems (Prasanna et al. 2013b; Chittapun et al. 2018; Álvarez et al. 2020). Nevertheless, these results are context-dependent: reductions in fertilizer inputs or production costs observed for a particular crop and cyanobacterial inoculant should not be assumed to be replicable across crop species or under different soil, climatic, and management conditions. Consequently, such advantages should be considered within the particular experimental and agronomic frameworks in which they were obtained, rather than being generalized to broader agricultural systems.

Biological N₂ fixation is a key functional trait of diazotrophic cyanobacteria and represents a potential source of nitrogen in agricultural systems. Under low combined-N availability, the oxygen-sensitive nitrogenase complex reduces atmospheric N₂ to ammonium (Mishra et al. 2019). Cyanobacteria protect nitrogenase by temporally separating fixation from oxygenic photosynthesis or by compartmentalizing it within heterocysts, where photosystem II is suppressed, and respiratory O₂ consumption helps maintain a microoxic environment (Fujita and Uesaka 2022). Fixation rates nevertheless vary by strain, light regime, oxygen dynamics, and carbon and phosphorus availability, reflecting the process’s high ATP and reducing-power requirements (Forchhammer and Selim 2020; Cheng et al. 2026).

Fixed ammonium is assimilated through the glutamine synthetase–glutamate synthase pathway. In heterocyst-forming filaments, reduced nitrogen is transferred to vegetative cells, while carbon skeletons move toward heterocysts to support nitrogenase activity (Bolay et al. 2018; Forchhammer and Selim 2020). Plants may access cyanobacterial N through direct symbiotic transfer, extracellular release, cell turnover, or mineralization of cyanobacterial biomass, with the relative contribution of each route varying among systems (Navarro et al. 2025). Beyond these direct or biomass-mediated inputs, cyanobacterial activity can influence the retention and cycling of N already present in the soil. Reported reductions in NH₃ volatilization may reduce gaseous N losses, while incorporating available N into microbial biomass can temporarily retain N within the soil microbial pool (Song et al. 2021). Subsequent microbial turnover and remineralization can return this N to mineral forms available for plant uptake. These retention and recycling processes may therefore improve soil-N conservation but should not be interpreted as evidence of direct transfer of newly fixed cyanobacterial N to plants (Song et al. 2021, 2022).

Growth responses associated with diazotrophic cyanobacteria have been reported in cotton inoculated with native soil strains, soybean receiving *Anabaena*–rhizobia consortia, and rice colonized by *Nostoc punctiforme* (Prasanna et al. 2014; Álvarez et al. 2020; Jiménez-Ríos et al. 2024). Increased N availability may support chlorophyll formation, photosynthesis, protein synthesis, and biomass accumulation, but these effects can overlap with phytohormonal, microbial, and soil-mediated processes (Navarro et al. 2025). In chickpea, *Anabaena laxa*-based inoculation increased yield, nodulation, root growth, and plant-N accumulation in the evaluated systems (Bidyarani et al. 2016; Prasanna et al. 2017). In field experiments, the combined application of *A. variabilis* SCAU30 (species currently classified as *Trichormus variabilis*) and 50% chemical fertilizer increased rice yields by 13.9%- 22% compared with the conventional fertilizer treatment (Bao et al. 2021). Rice responses to *A. torulosa* also varied with genotype and atmospheric CO₂ conditions, reinforcing the importance of crop–environment context (Bhardwaj et al. 2025). Increased biomass or yield should therefore not be attributed exclusively to N₂ fixation unless transfer of newly fixed N is directly quantified.

Cyanobacteria can also increase the availability of phosphorus and micronutrients. Application of *Aphanothece* sp. extracts increased N, P, and K uptake in tomato, although the chemically complex extract prevents attribution of the response to a single nutritional or signaling mechanism (Mutale-Joan et al. 2020). Phosphate mobilization may involve organic acids and phosphatases, whereas siderophores can increase iron (Fe) solubility and acquisition under limiting conditions (Yandigeri et al. 2011; Toribio et al. 2020; Afkairin et al. 2021; Nawaz et al. 2024). Because phosphorus is required for ATP generation and nitrogenase function, P acquisition is physiologically linked to N₂ fixation (Cheng et al. 2026). A hydroxamate siderophore produced by *Synechococcus mundulus* increased Fe uptake and growth-related traits in maize seedlings under hydroponic Fe deficiency, although field performance remains untested (Brick et al. 2025). Cyanobacterial inoculation has also been associated with higher Zn, Fe, Mn, and Cu concentrations and altered defense-enzyme activities in wheat and maize, although the contributions of plant physiology, soil chemistry, and associated microorganisms remain difficult to separate (Rana et al. 2012; Prasanna et al. 2015a).

Beyond specific nutrient-mobilization mechanisms, cyanobacterial biomass supplies organic carbon and nutrients during decomposition, stimulating microbial activity and affecting nutrient retention and gradual release (Maqubela et al. 2009; Do Nascimento et al. 2019). Processed extracts of *Nostoc* sp. PCC 7120 (reported as *Trichormus* sp. in the original study) containing ammonium, P, K, amino acids, and soluble carbohydrates also supported early crop growth in hydroponic assays, illustrating the nutritional potential of cyanobacterial biomass (Haraguchi and Shimizu 2025). Nonetheless, this requires validation in soil and field conditions. Overall, N₂ fixation, nutrient release from biomass, phosphate mobilization, metal chelation, and microbiome-mediated turnover operate simultaneously, and their relative contributions should be counted under defined crop–soil–environment combinations.

### Modulation of soil physicochemical properties

In recent years, cyanobacteria have emerged as promising agents for rehabilitating degraded soils and mitigating desertification, particularly in arid and semi-arid environments (Lan et al. 2014; Rossi et al. 2017). A central mechanism underlying these benefits is the production of extracellular polymeric substances (EPS), chemically complex, predominantly polysaccharide matrices. Cyanobacterial EPS are generally composed of heteropolysaccharides containing neutral sugars, such as glucose, galactose, xylose, and rhamnose, together with uronic acids and non-carbohydrate components, including proteins and varying proportions of lipids and extracellular DNA (Pereira et al. 2009; Xu et al. 2013). Their anionic character, associated with uronic acids and charged substituents such as sulfate groups, contributes to interactions with mineral surfaces and ions, while their hydrophilic polymeric structure promotes water retention (Muñoz-Rojas et al. 2018; Joshi et al. 2020). Cyanobacterial filaments and EPS interact directly with soil particles, promoting coating, enmeshment, and binding processes that increase aggregate stability and alter soil micromorphology (Malam Issa et al. 2007; Maqubela et al. 2009; Chamizo et al. 2018). These biochemical properties contribute to aggregate formation, carbon accumulation, water retention, and overall soil stability.

Cyanobacterial species differ in their effects on soil properties. *Scytonema javanicum* increased organic carbon and nitrogen pools, whereas *Phormidium ambiguum* produced more EPS and affected penetration resistance (Chamizo et al. 2018). These contrasts may reflect differences in nitrogen fixation, biomass production, and EPS allocation, but soil physicochemical properties, including texture, moisture, organic matter content, bulk density, pH, and salinity, strongly influence their agronomic relevance. Reported gains in water retention were greatest in sandy and loamy soils, although values cannot be generalized across formulations or experimental conditions (Adessi et al. 2018; Nisha et al. 2018; Asghari et al. 2022).

As the primary colonizers of soil biocrusts, cyanobacteria serve as microbial habitat engineers by promoting carbon and nitrogen accumulation and supporting heterotrophic colonization, ecological characteristics that underpin their increasing use as inoculants to improve soil fertility and structure in agricultural systems (Maqubela et al. 2009; Liu et al. 2011; Lan et al. 2013). Additionally, in rice soils (typically paddy soils), cyanobacteria play a crucial role in sustaining rhizosphere microbial structure and regulating carbon and nitrogen cycling genes (Priya et al. 2015; Iniesta-Pallarés et al. 2023; Kour et al. 2024). Cyanobacterial application increased soil phosphomonoesterase, invertase, and dehydrogenase activities, indicating enhanced microbial activity and nutrient cycling (Nisha et al. 2018). In particular, increased phosphomonoesterase activity promoted the conversion of organically bound P into available forms, while cyanobacterial treatment enhanced N mineralization, increasing soil N availability (Nisha et al. 2018). These effects are further reinforced by cyanobacteria-based formulations of *Anabaena torulosa* and *Calothrix* sp., which markedly increase glomalin-related soil proteins, extracellular polysaccharides, and soil dehydrogenase activity, promoting soil aggregation, organic carbon sequestration, and sustained microbial activity (Prasanna et al. 2016). These observations collectively support improved soil structure, nutrient cycling, and long-term agroecosystem stability through cyanobacterial use.

Soil physicochemical properties, including texture, salinity, pH, electrical conductivity, organic matter, land use, and crop species, are major determinants of cyanobacterial community composition and establishment in agricultural soils (Alghanmi and Jawad 2019). Beyond these abiotic factors, plant-related characteristics such as crop identity and root exudate composition further shape plant-associated cyanobacterial communities, highlighting the importance of considering both soil conditions and host plants when selecting effective inoculants (Ali and Glick 2024; Compant et al. 2025).

### Metabolite-driven plant growth promotion

**Fig. 1:**
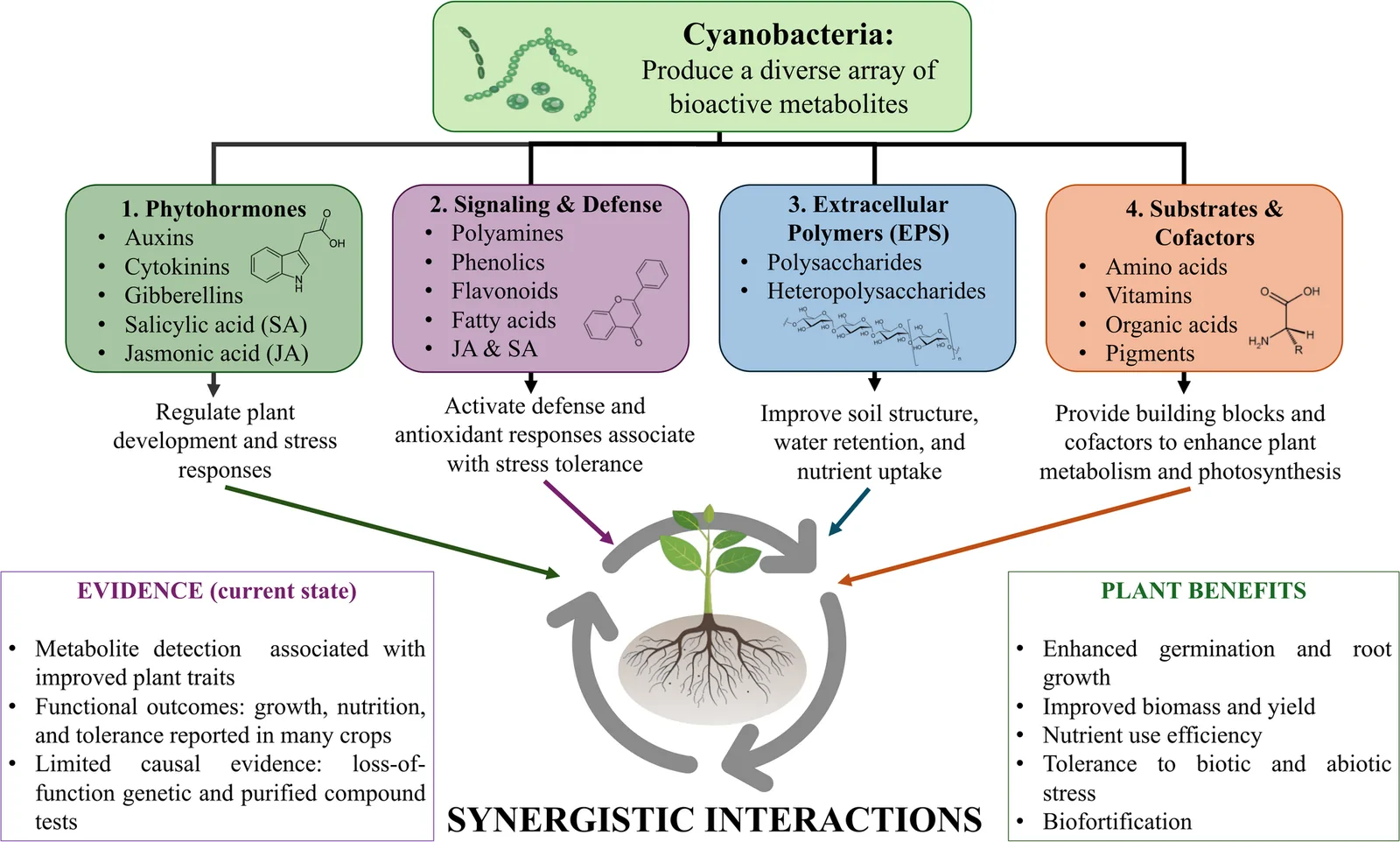
Mechanisms of cyanobacteria-derived metabolites in plant growth promotion and stress resilience

Cyanobacteria produce phytohormone-related compounds, amino acids, vitamins, proteins, pigments, and polysaccharides associated with germination, biomass, nutrient content, yield, and antioxidant responses (Singh 2014; Toribio et al. 2020; Kollmen and Strieth 2022; Abo-Shady et al. 2023; Nawaz et al. 2024). Evidence is strongest when a specific compound or pathway has been experimentally linked to a plant response (Fig. 1) (Hashtroudi et al. 2013; Mazhar et al. 2013; Zarezadeh et al. 2020; Gr et al. 2021; Jiménez-Ríos et al. 2024).

Auxins regulate root initiation, tropisms, apical dominance, and fruit development (Gomes and Scortecci 2021; Zhang et al. 2022; Batista-Silva et al. 2024). IAA and related compounds have been detected across *Anabaena*, *Arthrospira*, *Chroococcidiopsis*, *Leptolyngbya*, *Nostoc*, *Oscillatoria*,* Phormidium*, *Synechocystis*, and *Wollea*, although production may depend on L-tryptophan availability (Hussain and Hasnain 2011; Hashtroudi et al. 2013; Mazhar et al. 2013; Zarezadeh et al. 2020; Zhou et al. 2020). In *Nostoc* sp. OS-1, *ipdC* gene disruption reduced IAA production, root colonization, and growth promotion in rice and wheat, whereas genetic complementation of the mutant with a functional *ipdC* gene restored these traits, providing rare genetic evidence for a causal link between cyanobacterial IAA production and plant response (Hussain et al. 2015).

Cyanobacteria can also produce cytokinin-related compounds, including isopentenyladenine, zeatins, ribosides, and conjugates (Hussain and Hasnain 2011, 2012; Kimura et al. 2020; Zhou et al. 2020; Uniyal et al. 2022). Cytokinin production has been associated with growth, biomass, grain weight, nutrient content, and increased seed protein accumulation in several crops, including cotton, cucumber, garden geranium, pea, tomato, watercress, and wheat, although direct functional and causal tests remain limited (Osman et al. 2010; Hussain et al. 2010; Tejada-Ruiz et al. 2020; Toribio et al. 2020; Jiménez-Ríos et al. 2024). Gibberellin-related activity has also been reported in biostimulating species such as *Anabaena cylindrica*,* Limnospira platensis* (reported as *Arthrospira platensis* in older literature), *Nostoc* sp., and *Oscillatoria angustissima* (Osman et al. 2010; Gheda and Ahmed 2015; Ertani et al. 2019; Bayona-Morcillo et al. 2020; Tejada-Ruiz et al. 2020; Jiménez-Ríos et al. 2024).

Salicylic acid (SA) is a phenolic phytohormone that regulates plant growth, development, and immune responses against pathogens and abiotic stress (Khan et al. 2012; Myers et al. 2023; Kanwal et al. 2024). For instance, many cyanobacterial genera have been reported to produce salicylic acid, such as *Anabaena* sp. *Limnospira platensis*, *Calothrix* sp., *Dolichospermum* sp., *Gloeocapsa* sp., *Leptolyngbya* sp., *Nostoc* sp., *Nodularia* sp., *Tolypothrix* sp., and *Trichormus* sp. (Tejada-Ruiz et al. 2020; Toribio et al. 2020; Zhou et al. 2020; Jiménez-Ríos et al. 2024). However, direct experimental evidence linking cyanobacteria-derived salicylic acid to plant responses remains limited. Current studies do not distinguish its specific contribution from that of other bioactive compounds present in cyanobacterial cells or extracts. Consequently, the contribution of cyanobacteria-derived SA to plant growth, stress tolerance, or defense cannot yet be distinguished from the effects of other cyanobacterial metabolites. Another important phytohormone that activates plant defense mechanisms against pathogens and other stresses, such as salinity, temperature, and heavy metal stress, is the fatty acid jasmonic acid, which was identified in the hydrolysate of *Limnospira platensis* (Bayona-Morcillo et al. 2020), although it has not yet been functionally validated.

Many other enzymes and metabolites can act as biostimulators, reflecting improved photosynthetic and nutritional performance, nitrogen use efficiency, and antioxidant activity. Studies in maize and rice with these inoculants have shown a positive correlation between micronutrient mobilization (zinc, copper, iron, and manganese) in leaves/grains and phenylalanine ammonia-lyase and polyphenol oxidase activities (Adak et al. 2016; Prasanna et al. 2015a). Fatty acids in the extracts or released by cyanobacteria might activate these enzymes, which serve as elicitors (Gayathri et al. 2017). Other associative studies also attribute the plant growth-promoting and enhanced plant signaling ability to the presence of polyamines (such as spermine and spermidine) in the hydrolysate and the improved capacity of inoculated plants to accumulate phenylpropanoids, phenolic acids (gallic, gentisic, chlorogenic, and ferulic acids), and flavonoids (rutin and quercetin) (Singh et al. 2011; Adak et al. 2016; Mógor et al. 2018a).

Cyanobacterial cells and exudates contain amino acids, vitamins, organic acids, pigments, and polysaccharides that may support plant metabolism and defense (Singh 2014; Gr et al. 2021; Santini et al. 2021; Abo-Shady et al. 2023). *Limnospira platensis* biomass enhanced chlorophyll synthesis, leaf amino acids and proteins, and hypocotyl growth in red beet (Mógor et al. 2018b), whereas phycocyanin extracted from *L. platensis* induced capsaicin and anthocyanin accumulation in *Capsicum frutescens* and *Daucus carota*, respectively, providing functional evidence for a direct role of this cyanobacterial pigment in plant metabolic responses (Rao et al. 1996). Polysaccharides extracted from *Phormidium tenue* biomass applied at 2–240 mg L⁻¹ on seeds promoted seed germination and ion uptake in *Caragana korshinskii*, and subsequent exposure of three-leaf seedlings to these polysaccharides affected ROS and MDA levels and SOD activity, supporting reduced oxidative damage and enhanced antioxidant responses (Xu et al. 2013). Cyanobacterial exudates have also been shown to alter plant metabolic and stress-related responses, including proline accumulation, antioxidant enzyme activity, soluble sugars, and glutamine synthetase activity (Roque et al. 2023).

These effects are context-dependent and may arise from interactions among nutrients, signaling molecules, and changes in plant or microbial metabolism. Most studies report metabolite detection together with a plant response rather than purified-compound, loss-of-function, or dose–response evidence. Accordingly, most available evidence falls within the associative category, whereas functional and especially causal validation remains comparatively rare. The *ipdC* disruption/complementation experiments in *Nostoc* sp. OS-1 (Hussain et al. 2015) represent an important exception, illustrating the level of mechanistic resolution required to confidently assign a specific cyanobacterial metabolite to a plant association and phenotype. Mechanistic claims should therefore distinguish association from demonstrated causality.

### Cyanobacterial metabolites in plant protection and stress tolerance

#### Biotic stress mitigation: antimicrobial and signaling compounds

Cyanobacteria can protect plants against biotic stress, serving as a tool for pest and disease biocontrol. A wide range of cyanobacterial metabolites has been reported to exhibit antibacterial, antifungal, antiviral, and insect- and nematode-inhibitory activities (Demay et al. 2019; Kumar et al. 2019; Poveda 2021; Righini et al. 2020; Kar et al. 2022; Cock and Cheesman 2023). Numerous compound classes can be associated with these biological activities, including alkaloids, depsipeptides, lipids, lipopeptides, macrolides/lactones, peptides, polysaccharides, polyketides, and terpenes, among others (Weiss et al. 2025a). Considering the genetic potential for modifications achieved within biosynthetic pathways (e.g., NRPS - non-ribosomal peptide synthase, PKS - polyketide synthase, RiPPs - ribosomally synthesized and post-translationally modified peptides, and terpenes), the diversity and complexity of the cyanobacterial metabolome are greater and represent an unexplored source of biotechnology (Dittmann et al. 2015; Righini et al. 2022; Machado et al. 2025).

The secondary metabolites of cyanobacteria can exert direct inhibitory effects against a wide range of plant pathogens (Table 2). However, much of the evidence remains based on crude extracts, and only a limited number of compounds have been functionally validated. These effects of cyanobacterial extracts and filtrates are associated with chemically diverse compounds that interfere with essential cellular processes, including cell membrane integrity, enzymatic activity, cytoskeletal dynamics, cytotoxicity, quorum sensing, and signal transduction pathways (Kar et al. 2022). For example, aqueous extracts of *Anabaena oryzae* inhibited the root-knot nematode *Meloidogyne incognita* in banana, reducing the final population of nematodes in the soil (52.6%), an effect associated with an increase in phenolic-related metabolites in the banana roots (Hamouda and El-Ansary 2017). These compounds are thought to disrupt nematode metabolism, reducing infectivity and the number of juveniles in the soil. Similarly, fatty acid- and aliphatic hydrocarbon-rich extracts from *Nostoc piscinale* and *Anabaena variabilis* suppressed *Rhizoctonia solani* in rice, likely by destabilizing membranes and inhibiting fungal growth (Zhou et al. 2020).

Phenolic compounds, polysaccharides, and alkaloids have been associated with the antifungal activity of cyanobacteria. Culture filtrates from *Nostoc muscorum* and *Oscillatoria* spp. inhibited *Alternaria porri* in onion, while phenolic- and polysaccharide-containing extracts from *Nostoc entophytum* and *N. muscorum* reduced *Rhizoctonia solani* infection in soybean (Osman et al. 2011; Abdel-Hafez et al. 2015). Although these studies support an association between cyanobacterial metabolites and pathogen suppression, the contribution of individual compounds remains unresolved. Phenolics and other bioactive metabolites may affect fungal development by inhibiting spore germination, disrupting membrane and redox homeostasis, and interfering with metabolic pathways (Naji et al., 2017; Qi et al. 2023). More specialized compounds, including phycobiliproteins from *Limnospira platensis*, have also shown strong antifungal activity against *Botrytis cinerea* in tomato fruits, suggesting additional roles for light-harvesting proteins in suppressing biotic stress (Righini et al. 2020).

Evidence from more agronomically relevant systems further supports the biocontrol potential of cyanobacterial inoculants. In cotton and tomato, soil application of *Anabaena torulosa*, *Calothrix* sp., *Anabaena laxa*, and *A. variabilis* reduced diseases caused by *R. solani* and *Fusarium* sp., with hydrolytic enzymes and rhizosphere modulation proposed as contributing mechanisms (Prasanna et al. 2013a, 2016). However, these mechanisms remain associative rather than causally demonstrated. Similarly, cyanobacterial inoculation reduced rice sheath blight under microarea conditions across two growing seasons, while complementary in vitro assays identified several fatty acids and aliphatic hydrocarbons with antifungal activity against *R. solani* (Zhou et al. 2020).

Several cyanobacterial bioactive metabolites, identified in purified or partially purified fractions, have shown herbicidal or strong antifungal activity, including fischerellin A, cryptophycins, and balticidins (Hagmann and Jüttner 1996; Biondi et al. 2004; Bui et al. 2014). These findings provide functional evidence linking specific cyanobacterial metabolites to inhibitory biological effects, although further purification and compound-specific validation may be required depending on the study. Their potent bioactivity also raises concerns regarding environmental toxicity, selectivity, and regulatory compliance that must be addressed before agricultural application (Marrez and Sultan 2016).

**Table 2:** Bioactivity of cyanobacteria against plant pathogens and agricultural pests, with supporting experimental evidence^1^ related to the potential metabolites. N/A = not available or not reported

Crop	Pathogen/Pest	Cyanobacteria	Methods of application	Key results	Evidence ^1^	Potential Metabolites	References
Banana (*Musa* sp.)	*Meloidogyne incognita*	*Anabaena oryzae*	Cell culture	Reduction of 52.6% in the number of J2 in the soil	Associative	Phenolics compounds	Hamouda and El-Ansary (2017)
Cotton (*Gossypium hirsutum*)	*Rhizoctonia solani*	*Anabaena torulosa*, *Calothrix* sp.	Soil inoculation	Inoculants reduced the population of *R. solani*	Associative; Translational	Hydrolytic enzymes	Prasanna et al. (2016)
	*Spodoptera littoralis*	*Anabaena flos aquae*	Intracellular extract	Adult emergence decreased to 59.5%, and egg fertility decreased to 78.4%	N/A	N/A	Abdel-Rahim and Hamed (2013)
Fava bean (*Vicia faba*)	*Alternaria. alternata*,* Botrytis fabae*,* Fusarium oxysporum*,* F. solani*,* Macrophomina phaseolina*,* Rhizoctonia solani*,* Sclerotium rolfsii*	*Anabaena subcylindrica*,* Nostoc muscorum*,* Oscillatoria angusta*	Cell-free culture filtrate	*A. subcylindrica* filtrate caused inhibition in all fungi at 75% concentration	N/A	N/A	Abo-Shady et al. (2007)
Onion (*Allium cepa*)	*Alternaria porri*	*N. muscorum*,* Oscillatoria* sp.	Cell-free culture filtrate	Reduction of the linear growth of *A. porri* by 20.37 and 36.34%, and the disease severity by 55.1–66.5%	Associative	Phenolic compounds and alkaloids	Abdel-Hafez et al. (2015)
Rice (*Oryza sativa*)	*Rhizoctonia solani*	*Nostoc piscinale*,* Anabaena variabilis*	Dichloromethane/ethyl acetate cell extract	Inhibition of > 80% of rice sheath blight and mycelial growth (78.33–90.12%)	Associative; Translational	Fatty acids, aliphatic hydrocarbons	Zhou et al. (2020)
	*Xanthomonas oryzae*	*Anabaena variabilis* SCAU30	Volatile compounds	Efficacy against rice sheath blight and bacterial blight was 62.3% and 45.3%, respectively.	N/A	N/A	Bao et al. (2021)
Soybean (*Glycine max*)	*Rhizoctonia solani*	*Nostoc entophytum* and *N. muscorum*	Acetone, chloroform, methanol, and aqueous extracts	The antifungal activity of the water extract of *N. entophytum was* 44.4%	Associative	Phenolics and polysaccharides compounds	Osman et al. (2011)
Sugar beet (*Beta vulgaris*)	*Fusarium verticillioides*,* Rhizoctonia solani*,* Sclerotium rolfsii*	*Phormidium fragile* and *Nostoc muscorum*	Cell-free culture filtrate	Inhibition of fungal biomass (31–52%) and spores	N/A	N/A	Rizk (2006)
Tomato (*Solanum lycopersicum*)	*Fusarium* *oxysporum*,* F. moniliforme*	*Anabaena laxa*,* A. variabilis*	Soil inoculation	Increase of 8–29% and 8–10% for chitosanase and β-1,4-glucanase activity	Associative	Hydrolytic enzymes	Prasanna et al. (2013a)
	*Meloidogyne arenaria*	*Oscillatoria chlorina*	Cyanobacterial powder	Root galling and nematode population decreased by 68.9% and 97.6%	N/A	N/A	Khan et al. (2007)
	*Botrytis cinerea*	*Limnospira platensis* BEA 0007B [reported as *Arthosprira]*	Purified phycobiliproteins	Reduced disease severity by an average of 72.4%	Functional	Phycobiliproteins	Righini et al. (2020)
	*Clavibacter michiganensis*	*Leptolyngbya sp.*	Biomass crude extract	Inhibitory effect of 17% and foliar application reduced the infectivity by 50%	N/A	N/A	Toribio et al. (2021a)
Bean (*Phaseolus vulgaris*), Barley (*Hordeum vulgare*), Tomato, and Rice	*Uromyces appendiculatus*,* Erysiphe graminis*,* Phytophthora infestans*,* Pyricularia oryzae*	*Fischerella muscicola* UTEX 1829	Purified compound	Growth inhibition higher than 80% against fungi	Functional	Fischerellin A	Hagmann and Jüttner (1996)
Tomato, Chili (*Capsicum annuum*), and Eggplant (*Solanum melongena*)	*Pythium aphanidermatum*	*Calothrix elenkenii*	Cell-free culture filtrate and ethyl acetate extract	MIC values indicated that 850 ppm of culture filtrate and 16.6 ppm of the extract were inhibitory	N/A	N/A	Manjunath et al. (2010)
in vitro	*Fusarium oxysporum*,* Penicillium expansum*,* Phytophthora cambivora*,* P. cinnamomi*,* Rhizoctonia solani*,* Rosellinia sp.*,* Sclerotinia sclerotiorum*,* Helicoverpa armigera*	*Nostoc* sp. ATCC 53,789	Methanolic extracts	Biomass extract inhibited fungal growth at 0.25 g liter^− 1^. *H. armigera* larvae were killed at a dose of 2.20 mg cm^− 2^, with a 50% lethal concentration of 0.44 mg cm^− 2^	Associative	Cryptophycin	Biondi et al. (2004)
	*Aspergillus flavus*,* A. niger*,* Colletotrichum musae*,* Fusarium oxysporum*, and *Paecilomyces lilacinus*	*Oscillatoria*,* Phormidium*,* Synechococcus*,* Synechocystis*,* Trichodesmium*	Aqueous, methanol, n-propanol, and petroleum ether extracts	Methanol extracts showed 34.9% inhibition, followed by *n*-propanol (30.2%), petroleum ether (18.6% ), and water (16.2%)	N/A	N/A	Pawar & Purani (2008)
	*Fusarium oxysporum*	*Anabaena fertilissima* RPAN1	Cell-free culture filtrate	70% greater antifungal activity than the control	Associative	Chitosanase	Gupta et al. (2010)
	*Aspergillus flavus*,* A. parasiticus*,* A. westerdijikia*,* A. steynii*,* A. ochraceus*,* A. carbonarius*,* Fusarium verticillioides*,* F. proliferatum*,* Penicillium* *verrucosum*	*Microcystis aeruginosa*	Semi-purified diethyl ether extracts	Inhibition zone ranged from 10.3 to 21.6 mm	Associative	Hydroxytoluene (BHT) and Hexadecanoic acid	Marrez and Sultan (2016)
	*Meloidogyne incognita*,* M. graminicola*,* Heterodera cajani*,* H. avenae*,* Rotylenchulus reniformis*	*Synechococcus nidulans* CCC 464	Cell extract	Extracts caused immobility (91.3–98.4%) and mortality (17.4–31.5%) of J2.	N/A	N/A	Holajjer et al. (2012)
	*Fusarium moniliforme*,* Alternaria solani*,* Pythium aphanidermatum*,* Aspergillus candida*,* Drechslera oryzae*,* Fusarium solani*	*Anabaena sp*	Cyanobacterial cultures and culture filtrates	Zone of inhibition of varying diameter, ranging from 10 to 40 mm	Associative	Hydrolytic enzymes (chitosanase, endoglucanase, glucosidase)	Prasanna et al. (2008)
	*Aspergillus fumigatus*,* Mucor* sp.	*Anabaena cylindrica* Bio33	Purified compound	Inhibition zones from 21 to 32 mm	Functional	Balticidins A–D	Bui et al. (2014)

^1^Evidence categories follow the framework described in Sect. 5. Associative (A): whole cells, biomass, culture filtrates, crude extracts, or other chemically unresolved preparations produce a biological response, but the specific active mediator is not functionally validated. Functional (F): an isolated compound or chemically defined fraction is directly tested and reproduces the biological effect under controlled conditions. Causal (C): targeted genetic or biochemical perturbation alters the phenotype, with causality further supported, where available, by complementation, rescue, or metabolite add-back. Translational (T): efficacy is demonstrated under field or agronomically relevant conditions. A, F, and C indicate increasing levels of mechanistic support, and each entry is assigned the highest level supported by the reported evidence. T is an orthogonal descriptor and may accompany A, F, or C. N/A indicates that evidence grading is not applicable to the information or experimental design represented by the entry.

Beyond direct antagonism, microbial inoculation or metabolite application activates key defense-related enzymes and pathways associated with induced systemic resistance (ISR) and systemic acquired resistance (SAR), thereby enhancing plant growth, improving yields, and enhancing product quality (Singh 2014; Rabari et al. 2023). The effects of protecting and developing immune responses in tomato plants against pathogens using cyanobacterial extracts containing salicylic acid were studied, specifically in the presence of *Clavibacter michiganensis* subsp. *michiganensis* (bacterial canker) (Toribio et al. 2021a). Structurally diverse metabolites, including polysaccharides, phenolic compounds, fatty acids, amino acids, and proteins, can act as microbe-associated molecular patterns (MAMPs), triggering early defense signaling and priming plant immune responses (Lu et al. 2022; Karimi et al. 2025). Although the molecular mechanisms underlying these responses are still emerging, some evidence suggests that cyanobacterial-derived compounds might act as effective elicitors of plant immunity.

In tomato seedlings of a tripartite interaction involving *Fusarium* sp. and *Anabaena* sp., cyanobacterial formulations significantly enhanced the activity of phenylalanine ammonia-lyase (PAL) and polyphenol oxidase (PPO), enzymes central to phenylpropanoid metabolism and the synthesis of antimicrobial phenolics, as well as pathogenesis-related enzymes such as chitosanase and β-1,3-glucanase, which contribute to fungal cell wall degradation (Prasanna et al. 2013a; Priya et al. 2015). Similarly, in cotton plants infected with *Rhizoctonia solani*, cyanobacterial treatments increased β-1,3-endoglucanase and chitosanase activities, respectively, and markedly reduced pathogen population and disease severity (Prasanna et al. 2016). Furthermore, in nematode–plant systems, *Anabaena oryzae*, alone or in combination with other microalgae, reduced *Meloidogyne incognita* juvenile populations in banana plants while simultaneously increasing the accumulation of phenolic compounds and antioxidant metabolites in plant tissues (Hamouda and El-Ansary 2017). These biochemical changes underscore cyanobacteria as next-generation biostimulants and biocontrol agents through a dual mode of action: direct suppression of pathogens and activation of plant systemic defense responses, thereby strengthening cell wall-associated barriers and stimulating the production of antimicrobial and antioxidant metabolites.

#### Abiotic stress tolerance: lessons from extremophilic cyanobacteria

Drought and salinity responses to cyanobacterial treatments have been associated with EPS, osmolytes, antioxidants, pigments, hormones, and stress-related proteins (Yadav et al. 2022; Gr et al. 2021). Extremophilic cyanobacteria provide candidate traits for stress-tolerant formulations, but much of the evidence derives from cyanobacterial stress physiology rather than direct tests in crops. Plant responses also depend on strain, formulation, dose, crop genotype, and stress intensity (Abd El-Baky et al. 2010; Singh and Dhar 2010; Singh 2014; Gupta and Huang 2014; Li et al. 2019; Bayona-Morcillo et al. 2020; Tejada-Ruiz et al. 2020; Nawaz et al. 2024).

Cyanobacteria promote plant growth under drought by synthesizing exopolysaccharides that, in biological soil crusts, improve water and nutrient retention and protect microbial communities from desiccation (Colica et al. 2014; Adessi et al. 2018). By improving moisture retention around the seed within the spermosphere during germination and subsequently modifying the rhizosphere microenvironment, these processes may contribute to the beneficial effects of cyanobacterial seed priming, including increased germination and enhanced plant growth under drought stress (Maqubela et al. 2009; Muñoz-Rojas et al. 2018; Chua et al. 2020; Kollmen et al. 2025).

Plant adaptation to and tolerance of salinity stress, as well as osmotic balance, encompass complex physiological traits, metabolic pathways, and molecular or genetic networks (Gupta and Huang 2014). Improved performance of rice plants under saline conditions after inoculation with a cyanobacterial consortium (*Anabaena fertilissima*,* Anabaena anomala*, and *Nostoc muscorum*) was associated with reduced sodium (Na⁺) accumulation in plant tissues and increased chlorophyll and proline contents (Abbas et al. 2015). These responses were discussed in relation to cyanobacterial production of exopolysaccharides, phytohormones, and amino acids, which may collectively contribute to plant tolerance to salt stress. When introduced to saline-alkaline soil, *Nodularia* sp. lowered the pH from 8.34 to 7.57 and markedly increased the availability of nutrients, including nitrogen, phosphorus, and organic matter, over 5 months (Zheng et al. 2025). Halotolerant nitrogen-fixing cyanobacteria secrete extracellular polysaccharides, organic acids, and enzymes in response to salinity, thereby improving soil properties and promoting better plant development under saline conditions (Singh and Dhar 2010). In addition, indigenous cyanobacteria can enhance organic matter and modulate microbial activities (dehydrogenase, invertase, and phosphomonoesterase), thereby improving plant response to salinity stress through osmotolerance mechanisms (Nisha et al. 2018).

Researchers have also expressed cyanobacterial genes in plants to improve abiotic-stress tolerance. Examples include flavodoxin, DnaK, WspA, and metal- or salt-tolerance genes (Tognetti et al. 2006; Takabe et al. 2008; Xu et al. 2010; Ai et al. 2014). Researchers have modified crops such as tobacco (*Nicotiana tabacum*) and creeping bentgrass (*Agrostis stolonifera*) to express the flavodoxin (Fld) gene from *Anabaena* sp. PCC 7119, offering an alternative approach to mitigating drought stress by modulating carbohydrate and energy metabolism and oxidative stress responses (Tognetti et al. 2006; Gharechahi et al. 2015; Li et al. 2017). Interestingly, plants expressing the *fld* gene displayed increased tolerance to multiple stressors, including redox-cycling herbicides, N starvation, high temperatures, high irradiance, water deficit, and UV radiation. This transgenic strategy highlights useful cyanobacterial functions but differs from using cyanobacteria as biostimulants (Coba de la Peña et al., 2010; Cui et al. 2018).

### Cyanobacteria-mediated remediation and plant performance under contaminant stress

In contaminated agricultural environments, cyanobacteria can indirectly improve plant performance by reducing contaminant mobility, bioavailability, or toxicity. These effects arise from several remediation processes, including bioaccumulation, surface-mediated biosorption, metabolic transformation, and biodegradation of organic and inorganic contaminants (Mondal et al. 2019; Cui et al. 2021; Nawaz et al. 2024). In agricultural ecosystems, an important source of contamination is the extensive use of organic pollutants, including herbicides, fungicides, insecticides, and nematicides, which are essential to modern crop production but pose substantial ecological risks (de Souza et al. 2020; Goel et al. 2024).

Cyanobacteria can transform or adsorb selected pesticides, but responses vary by strain and compound. *Nostoc* sp. ATCC 27347 (reported as *Anabaena* sp.), *Limnospira fusiformis* CCALA 023 (reported as *Arthrospira fusiformes*), *Leptolyngbya boryana* ATCC 27894, *Microcystis aeruginosa* PCC 7941, *Nostoc punctiforme* ATCC 29133, and *Limnospira platensis* C1 (reported as *Spirulina platensis*) strains can use glyphosate-derived phosphorus (Forlani et al. 2008). Extracts of *Limnospira platensis* and *Nostoc muscorum* mitigated bromoxynil injury in wheat through antioxidant and detoxification responses (Gaafar et al. 2022). Cultures of cyanobacteria like *Synechocystis* sp. and *Phormidium* sp. show higher removal capacities for imidacloprid when grown in media supplemented with triacontanol and salicylic acid (Aminfarzaneh and Duygu, 2010). Furthermore, *Scytonema hofmannii* and *Fischerella* contributed to methyl-parathion removal (Tiwari et al. 2017), and chlorpyrifos degradation products were detected in rice associated with *Nostoc commune* MBDU 101 (Gayathri et al. 2024). These studies support remediation potential but not broad field efficacy.

Heavy metals represent a major class of environmental contaminants that negatively affect soil quality, plant development, and crop productivity (Rashid et al. 2023). In agricultural and industrially impacted environments, cyanobacteria have demonstrated a potential to bioremediate a wide range of toxic metals, including arsenic (As), cadmium (Cd), chromium (Cr), copper (Cu), lead (Pb), mercury (Hg), nickel (Ni), and zinc (Zn). Their remediation potential derives from a combination of extracellular and intracellular mechanisms that can reduce metal mobility, bioavailability, and toxicity in contaminated environments (Al-Amin et al. 2021).

Extracellular polymeric substances (EPS) provide functional groups that adsorb and immobilize metal ions and can alter their speciation and toxicity (Al-Amin et al. 2021). In contaminated biocrusts, *Nostoc commune* EPS production correlated with immobilization of copper, lead, and zinc (Pagli et al. 2024). Cyanobacteria also use phytochelatins, metallothioneins, transporters, antioxidants, and metal-transforming enzymes for intracellular detoxification (Chakdar et al. 2022). Genomic studies with cyanobacteria identify arsenic-resistance pathways involved in sequestration, reduction, methylation, and efflux (Miyashita et al. 2015; Xue et al. 2017). In arsenic-contaminated soils, *Leptolyngbya* sp. XZMQ reduced arsenic bioavailability and increased the abundance of the genes *aio*A, *arr*A, *ars*C, and *ars*M, linking inoculation to altered arsenic transformation at the microscale (Mao et al. 2023).

Beyond soil detoxification, cyanobacteria-mediated metal remediation can directly benefit plant performance. Seed priming with *Limnospira platensis* (reported as *Spirulina platensis*) has been shown to limit cadmium translocation from roots to shoots, enhance seed germination, and promote maize growth under Cd stress (Seifikalhor et al. 2020). Moreover, diverse cyanobacterial taxa, such as *Limnococcus* sp., *Nostoc muscorum*, and *Synechococcus* sp., have shown broad-spectrum metal-removal capacity, efficiently sequestering ions including copper, nickel, lead, cadmium, zinc, and cobalt (Al-Amin et al. 2021). Beyond direct cyanobacterial applications, the functional relevance of cyanobacterial metal-detoxification genes has also been demonstrated heterologously: *Arabidopsis thaliana* plants transformed with the *smtA* gene, which encodes a metallothionein from *Synechococcus* sp., showed enhanced zinc tolerance associated with increased reactive oxygen species (ROS)-scavenging activity (Xu et al. 2010).

Future methodological approaches should distinguish among biosorption, bioaccumulation, and biodegradation or biotransformation rather than relying solely on contaminant-removal endpoints (Ciani and Adessi 2023; Żymańczyk-Duda et al. 2022). Greater attention should also be given to immobilized biomass, biofilms, and cyanobacteria-based consortia, which represent promising formats for improving biomass recovery and operational applicability (Banerjee et al. 2015; Ciani and Adessi 2023). Long-term outdoor and pilot-scale studies are also needed to evaluate the reproducibility of remediation performance under variable environmental conditions and address operational constraints associated with scale-up and biomass management (Morillas-España et al. 2021; Ahmad 2022; Melo et al. 2022; Lourembam et al. 2024). Thus, bioremediation should be considered an indirect plant-beneficial function rather than a direct biostimulant mechanism, as its contribution to plant performance depends on reducing contaminant exposure or toxicity in the surrounding environment.

## From mechanisms to cyanobacterial biostimulants

Plant biostimulants include microbial agents and non-microbial bioactive substances, such as beneficial bacteria and fungi, humic compounds, seaweed extracts, protein hydrolysates, biopolymers, and inorganic elements (Yakhin et al. 2017; Garg et al. 2024; Arinaitwe et al. 2025). Cyanobacteria bridge these categories because living cells, biomass, and derived metabolites can affect plant nutrition, signaling, stress responses, and microbiome structure (Bibi et al. 2024; Sun et al. 2026).

Most studies assume that cyanobacterial inoculation possesses general growth-promoting traits, such as N₂ fixation, phosphate solubilization, phytohormone production, excretion of bioactive compounds, and symbiotic associations (Múnera-Porras et al. 2020). Nevertheless, these mechanisms likely represent only a fraction of cyanobacteria’s biostimulant potential, as they harbor an extensive repertoire of metabolites, signaling molecules, peptides, antioxidants, and other specialized compounds that may influence plant development and stress responses through yet unknown pathways. For example, extracellular polymeric substances enhance soil aggregation, water retention, and rhizosphere stability, thereby indirectly supporting plant hydration and nutrient uptake (Adessi et al. 2018). Osmolytes and compatible solutes contribute to abiotic stress tolerance by stabilizing cellular structures and redox homeostasis (Yadav et al. 2022). Meanwhile, fatty acids, phenolic compounds, and peptides exert antimicrobial activity or prime plant defense responses (Cock and Cheesman 2023). Pigments such as carotenoids, phycobiliproteins, and scytonemin further contribute to photoprotection, reduce soil temperature, and mitigate oxidative stress (Couradeau et al. 2016). However, biostimulants derived from cyanobacteria should be recognized as components with distinct biological functions, and products should address specific agricultural issues associated with these compounds’ bioactivity.

### Metabolomics as a tool for cyanobacterial biostimulant discovery

Cyanobacterial strains can be translated into commercial applications by identifying key bioactive compounds or functional traits (e.g., auxins, extracellular polysaccharides, or elicitor molecules), validating their causal effects on plant performance, and incorporating them into stable formulations, either as defined metabolite-based products or as optimized microbial consortia. Traditionally, researchers have evaluated cyanobacteria based on their overall effects on plant performance, with limited understanding of the compounds driving these responses, despite growing evidence for the diverse metabolites discussed in previous sections. Advances in metabolomics analyses play a central role by enabling comprehensive characterization of chemical diversity, identifying candidate bioactive metabolites, and prioritizing compounds potentially associated with plant growth promotion and stress resilience (Nephali et al. 2020; García et al. 2022). When integrated with functional genomics and formulation knowledge, this approach moves from descriptive observations to targeted, reproducible technologies, thereby enabling the development of next-generation biostimulants with scalable performance.

However, identifying candidate metabolites does not necessarily imply a causal relationship with plant responses. Most cyanobacterial compounds detected through untargeted metabolomic approaches remain putatively annotated, and their biological functions often require experimental validation (Campos et al. 2024; Machado et al. 2025; Weiss et al. 2025b). Zhou et al. (2020) provide an example: they first screened cyanobacterial strains for suppression of rice sheath blight and then validated selected strains under microarea conditions across two growing seasons. Chemical profiling of antifungal extracts by GC–MS then identified candidate metabolites, several of which were subsequently tested as analytical standards in dose–response assays against *Rhizoctonia solani*. This workflow illustrates how phenotypic screening can be integrated with metabolite profiling and functional assays to prioritize bioactive compounds. However, the causal contribution of these metabolites to disease suppression in the plants remains to be established.

Viewing cyanobacterial metabolites as functional elements opens new opportunities to design tailored biostimulant formulations that combine defined metabolite classes to target specific agronomic constraints. This perspective also highlights the importance of strain-species specificity, dosage, timing, and environmental context, as the same cyanobacterial species or compound may elicit distinct physiological responses depending on the plant species, developmental stage, or stress intensity (Zhang and Sonnewald 2017; Arinaitwe et al. 2025). Future studies should therefore combine metabolite isolation, structural confirmation, dose-response experiments, and mechanistic investigations to establish direct links between cyanobacterial metabolites and plant phenotypes.

### Delivery methods of cyanobacterial formulations

**Fig. 2:**
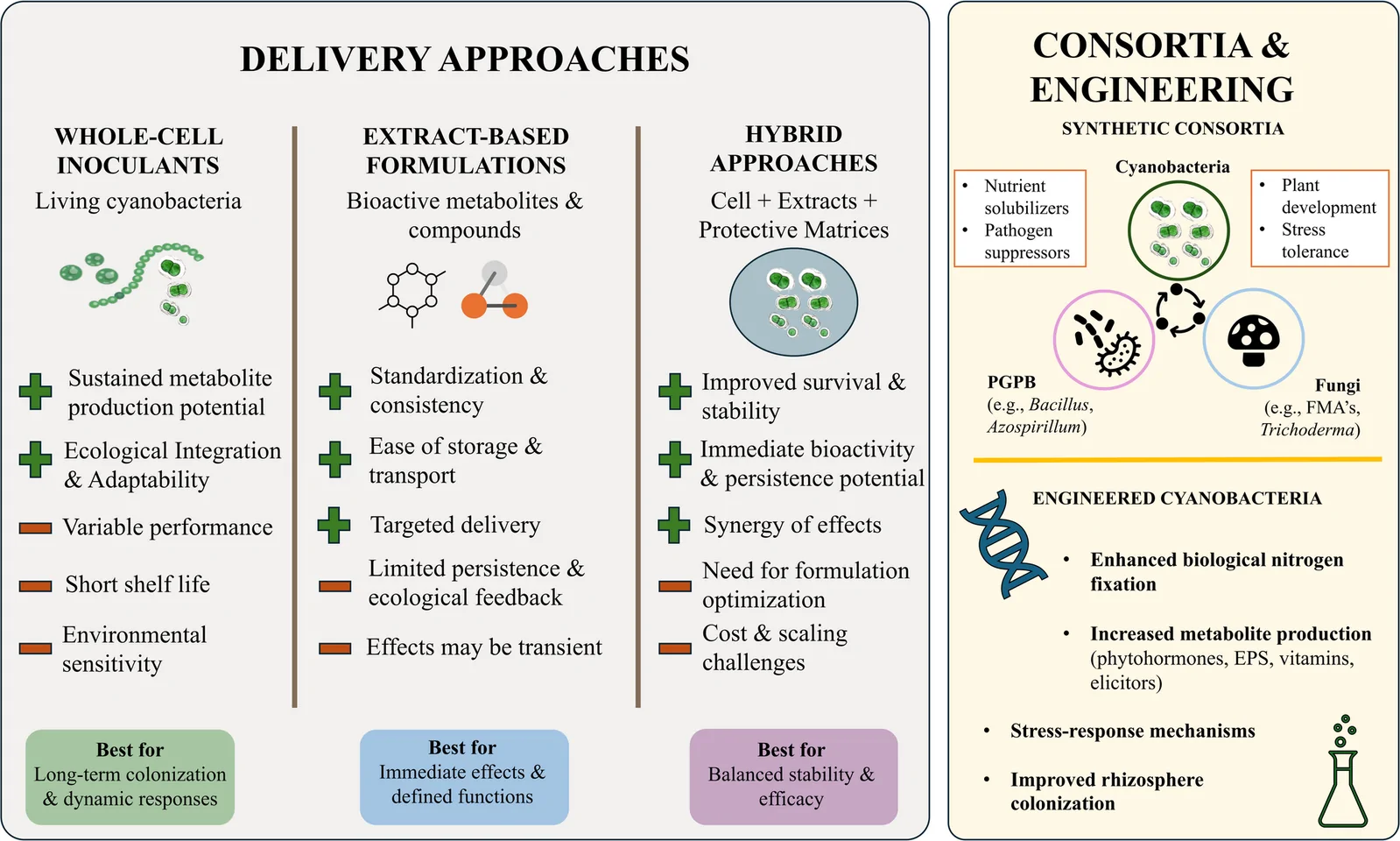
Overview of current and emerging strategies to enhance the agricultural application of cyanobacteria. Delivery approaches include whole-cell inoculants, extract-based formulations, and hybrid systems combining living cells with bioactive compounds. Future developments include synthetic microbial consortia and engineered cyanobacteria to improve plant growth promotion, stress tolerance, nutrient acquisition, and rhizosphere colonization

Whole-cell applications (living cyanobacteria) offer advantages such as sustained metabolite production, ecological integration, and the capacity to respond dynamically to environmental signals (Fig. 2). Biopriming seeds with cyanobacterial cultures has been shown to enhance germination rates and seedling development and to facilitate plant establishment by modulating physiological and biochemical responses in arid soils and under drought stress (Chua et al. 2020; Machado-de-Lima et al. 2023; Sneha et al. 2024). Cyanobacterial priming with *Anabaena torulosa* and *Anabaena laxa* produced robust nurseries, enhancing seedling emergence (up to 97.3%), increasing vigor by 23.6–43.8%, and stimulating enzymatic activity and rhizosphere development, with effects varying across tomato cultivars (Varsha et al. 2025). The inoculation of *Nostoc* sp. HNBGU006 in pot soil increased root length, shoot length, dry weight, and yield of *Allium sativum*. Additionally, a 1% aqueous extract enhanced various seedling growth parameters, demonstrating this cyanobacterial strain’s dual capacity as both an inoculum and a bioproduct (Uniyal et al. 2024).

However, the performance of cell-based cyanobacterial inoculations under field conditions is often inconsistent, reflecting multiple interacting constraints. Competition with native microbiota can limit the establishment and persistence of introduced strains, particularly when they lack ecological compatibility or a niche advantage. Environmental and edaphic factors directly influence cyanobacterial growth, metabolic activity, and survival, often reducing their effectiveness compared to controlled conditions. In addition, exposure to biotic pressures (e.g., grazing, pathogens) and chemical stressors (e.g., agrochemicals, salinity) can impair cell viability and functional performance. These limitations are further intensified by formulation challenges, including short shelf life, low stability during storage and transport, and difficulties in maintaining viable biomass at scale, as well as regulatory constraints associated with the approval and standardization of microbial products (Bauer et al. 2018; Santini et al. 2021; Arinaitwe et al. 2025).

Extract-based formulations are easier to standardize, store, and deliver than living cells, although efficacy and regulatory requirements remain product- and jurisdiction-specific (Fig. 2). Extracts or hydrolysates of *Arthrospira* and other cyanobacteria have improved growth, nutrient status, or stress responses in basil, beans, maize, cucumber, pumpkin, tomato, and wheat (Shariatmadari et al. 2013; Anitha et al. 2016; Mutale-Joan et al. 2020; Santini et al. 2022). Mixtures with non-cyanobacterial algae have also shown benefits, but researchers cannot attribute these effects to the cyanobacterial component alone (Shaari et al. 2025; De Moraes et al. 2026). Protein hydrolysate extracts prompted microbiome alterations, and certain benefits may partially arise from changes in the composition and activity of plant-associated communities (Colla et al. 2017). Nonetheless, these effects require temporal and functional validation, including repeated assessments throughout the crop cycle and, where relevant, across successive growing seasons to determine whether community shifts persist and how they relate to measurable functional changes. Recent evidence with *Limnospira indica* culture media and biomass extracts showed formulation- and application-dependent changes in tomato growth and rhizosphere/endosphere bacterial communities, linking extract composition and polysaccharide enrichment to microbiome modulation (Renaud et al. 2026).

Hybrid formulations combine protected living cells with rapidly available metabolites to balance biological activity, stability, and delivery (Fig. 2). Bentonite–sand pellets containing native *Nostoc*, *Scytonema*, and *Leptolyngbya* facilitated field dispersion and soil colonization, although declining viability during storage revealed stabilization constraints (Román et al. 2020). Alginate-based matrices enriched with cyanobacterial extracts promoted plant growth and protection against *Pythium ultimum* (Toribio et al. 2021b). Seed coatings based on carboxymethyl cellulose or gum arabic also improved early vigor, biomass, and rhizosphere structure and function compared with priming or drenching, illustrating the potential of integrated cell–extract delivery systems (Kumari et al. 2025).

Beyond single-strain applications, cyanobacteria are increasingly recognized as central components of synthetic microbial consortia designed to enhance plant performance (Prasanna et al. 2012, 2017; Álvarez et al. 2020). As phototrophic organisms capable of carbon and nitrogen fixation, cyanobacteria function as metabolic hubs that support heterotrophic partners involved in nutrient solubilization, phytohormone production, and pathogen suppression, reflecting natural microbial association (Fig. 2) (Maier et al. 2018; Pagli et al. 2025). Cyanobacterium-based bacterial and fungal biofilms have been shown to play a key role in integrated nutrient management in cotton cultivation, increasing seed cotton yield by 57–71%. Under the economic conditions evaluated in India, the combined seed-treatment and soil-drench application generated an additional income of US$462 ha⁻¹, while reduced nitrogen fertilizer use represented a separate saving of US$6.34 ha⁻¹ (Velmourougane et al. 2025). Nonetheless, the stability and reproducibility of synthetic consortia under field conditions remain major challenges due to ecological competition and environmental variability.

Consortia composed of cyanobacteria and PGPR (Plant Growth-Promoting Rhizobacteria) such as *Azospirillum* sp., *Azotobacter chroococcum*, *Bacillus* sp., *Brevundimonas diminuta*, *Mesorhizobium sp.*, *Rhizobium tropici*,* Pseudomonas* sp. (Horácio et al. 2025; Gavilanes et al. 2020; Prasanna et al. 2012, 2017; Rana et al. 2015; Solomon et al. 2023; Swarnalakshmi et al. 2013) significantly enhanced plant yield and defense mechanisms, while also improving nitrogen status through increased leghemoglobin content in nodules, improving symbiotic performance of the legumes and grasses, and promoting higher macro- and micronutrient accumulation in grains. In fungal biofilms, *Trichoderma* sp. also protects plants against pathogens, reducing plant mortality, while improving microbial community and plant productivity (Prasanna et al. 2015a, b; Kanchan et al. 2019). Beyond biofilm experiments, adding additives to cyanobacterial inoculations could enhance their biological activity. The foliar sprays comprising the combination of micronized *Lithothamnium* sp., a calcareous alga, and *Limnospira platensis* have been shown to enhance plant growth, stimulate plant metabolism, and increase the yield of *Allium cepa*; notably, the authors observed that biweekly applications represent a more cost-effective and environmentally friendly strategy for sustaining these yield gains using this approach (de Oliveira Amatussi et al. 2023).

Genome mining, metabolic modeling, CRISPR editing, synthetic circuits, and high-throughput screening are enabling pathway-guided biostimulant design (Behler et al. 2018; Garg et al. 2024; Navarro et al. 2025). Engineering strategies include enhanced metabolite production, stress-responsive regulation, rhizosphere competence, ammonium or amino-acid excretion, and expression of cyanobacterial nitrogen-fixation functions in plants (Chaurasia and Apte 2011; Thiel 2019; Liu et al. 2025; Verma and White 2025). Hormones, extracellular polymers, and peptide elicitors are additional targets. However, researchers must evaluate ecological safety, genetic stability, containment, and public acceptance before agricultural deployment.

### Safety considerations and cyanotoxin risk in agricultural applications

Cyanotoxin production is strain-dependent and cannot be predicted from genus or species identity alone. Toxigenic and non-toxigenic strains may occur within the same species, as shown for *Microcystis aeruginosa*, in which strains differ in microcystin production and physiological traits (Jacinavicius et al. 2019). Broader surveys also show substantial variation in toxin production among strains and taxa (Campos et al. 2024). Thus, the presence of toxigenic members within a genus does not mean all strains produce toxins, and the absence of reported toxigenicity should not be taken as evidence of safety. Strains intended for agricultural use should therefore be evaluated individually, with taxonomy serving as an initial guide rather than a substitute for toxicological screening. Importantly, when the original agricultural study did not evaluate cyanotoxin production, the toxicological status of the tested strain should be regarded as unknown rather than inferred from its taxonomic identity.

Safety assessment of strains intended for agricultural applications should combine molecular or genomic screening with chemical analysis. Genomic screening can target biosynthetic gene clusters associated with major cyanotoxin classes, including *mcy*, *nda*, *cyr*, *sxt*, and *ana*, involved in the biosynthesis of microcystins, nodularin, cylindrospermopsins, saxitoxins, and anatoxins, respectively (Dittmann et al. 2013; Pearson et al. 2016). Researchers have also characterized the *gnt* cluster responsible for guanitoxin biosynthesis (Lima et al. 2022). Detecting a cyanotoxin biosynthetic gene cluster indicates biosynthetic potential but does not demonstrate toxin production under conditions relevant to product manufacture or agricultural application. Conversely, failure to detect a limited set of known toxin genes does not establish toxicological safety, particularly considering the diversity of cyanobacterial secondary metabolites. Chemical analysis is therefore necessary to confirm toxin production and determine cyanotoxin concentrations (Chorus and Welker 2021).

Multi-class LC–MS/MS is particularly useful because it can screen several cyanotoxin groups in a single analysis. Jacinavicius et al. (2023a), for example, developed a method covering microcystins, nodularin, cylindrospermopsins, anatoxins, guanitoxin, and saxitoxin-related compounds. Nevertheless, the absence of detectable classical cyanotoxins should not be considered evidence of complete toxicological safety, since cyanobacteria also produce a broad range of bioactive metabolites that are not routinely monitored and remain poorly characterized (Jacinavicius et al. 2023b).

Risk also depends on the form in which cyanobacteria are applied. Whole-cell biomass and cyanobacterial extracts are not toxicologically equivalent. Many cyanotoxins, particularly microcystins, are predominantly intracellular in intact cells and may be released after cell damage or lysis (Chorus and Welker 2021). Products containing viable cells or intact biomass therefore require consideration of cell persistence, lysis, and subsequent release of intracellular metabolites. Crude or cell-free extracts represent a different exposure scenario because soluble metabolites may be more readily available to plants and soil organisms (Mohamed et al. 2022). Their composition depends on the extraction procedure and may include complex mixtures of known and unidentified bioactive compounds (Jacinavicius et al. 2023b). Biological effects observed with crude extracts cannot necessarily be attributed to a single metabolite or toxin class. Therefore, products derived from intact cells and extracts should be evaluated separately, based on their intended use.

Safety evaluation should also consider the fate of cyanotoxins after agricultural application. Cyanotoxins introduced into agricultural soils may interact with soil components, undergo degradation, persist for variable periods, or remain bioavailable for plant uptake (Weralupitiya et al. 2022). Experimental studies with microcystins and cylindrospermopsin demonstrate that crops can absorb these toxins and, depending on the toxin, crop, and exposure conditions, translocate them to aboveground or edible tissues (Corbel et al. 2014; Machado et al. 2017; Llana-Ruiz-Cabello et al. 2019). More recently, Van Hassel et al. (2025) demonstrated crop- and irrigation-dependent accumulation of microcystin-LR under mid-scale greenhouse conditions, including detection in lettuce leaves and strawberry fruits following spray irrigation. More directly relevant to cyanobacteria-based agricultural products, Massa et al. (2024) evaluated *Raphidiopsis raciborskii* (= *Cylindrospermopsis raciborskii*) biomass as a soil amendment for lettuce and demonstrated transfer of cylindrospermopsin to the soil–plant system. Although concentrations in edible tissues remained below the tolerable daily intake considered in that study, the results demonstrate that transfer from applied cyanobacterial biomass can occur. Safety assessment should therefore consider toxin persistence in soil, root uptake, translocation, and potential accumulation in edible plant tissues.

Current cyanotoxin guideline values have primarily been developed for drinking-water and recreational exposure rather than for cyanobacteria-based agricultural products or food crops. WHO health-based values for drinking water include provisional lifetime guideline values of 1 µg L⁻¹ for microcystins and 0.7 µg L⁻¹ for cylindrospermopsins, together with short-term or acute values for selected cyanotoxins (WHO, 2026). However, these values were derived for water exposure scenarios and should not be interpreted as regulatory limits for microbial biostimulants, biofertilizers, soil amendments, or edible plant tissues. Application-specific and harmonized thresholds for cyanotoxin occurrence in cyanobacteria-based agricultural products remain an important regulatory and research need (Chabili et al. 2024; Sun et al. 2026). Until such standards are established, existing health-based values (WHO guideline) may provide toxicological context, but safety assessment should be based on the intended product, application route, exposure scenario, and potential transfer through the soil–plant–food continuum.

## From evidence to validation for agricultural deployment

**Fig. 3:**
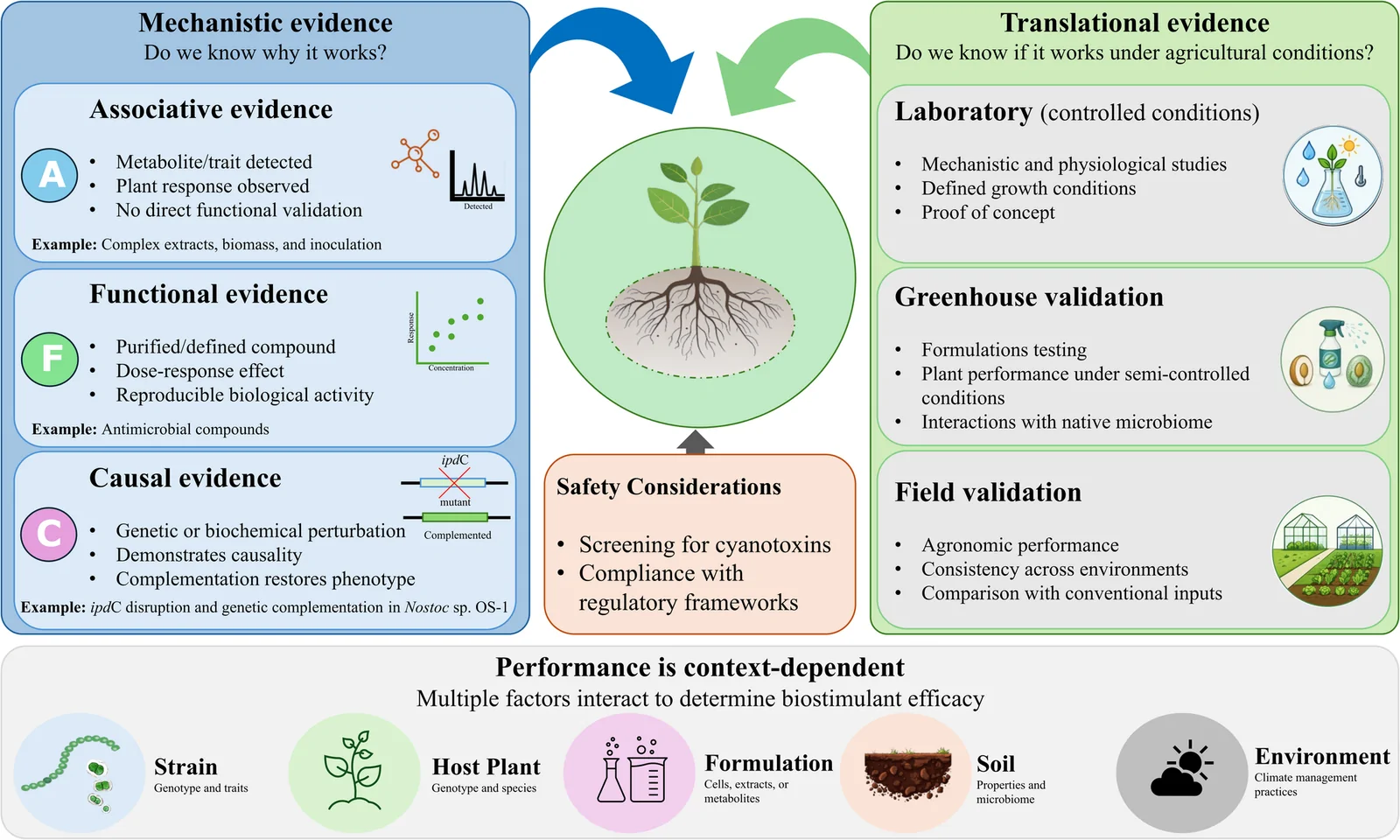
Conceptual framework for mechanistic and translational validation of cyanobacteria-based biostimulants. Mechanistic evidence may comprise associative evidence (A), in which metabolites or microbial traits coincide with plant responses without direct functional validation; functional evidence (F), based on defined compounds or treatments, dose–response relationships, and reproducible biological activity; and causal evidence (C), established through targeted genetic or biochemical perturbation. These categories are complementary rather than strictly sequential. In parallel, translational evidence evaluates performance from controlled laboratory experiments through greenhouse validation to field conditions. Safety assessment should accompany development throughout this process. Biostimulant performance is context-dependent and reflects interactions among cyanobacterial strain, host plant, formulation, soil properties and microbiome, and environmental and management conditions. Reliable cyanobacterial biostimulants therefore require integrating mechanistic support, agronomic reproducibility, and safety assessment within defined agricultural contexts

Translating cyanobacterial biostimulants into reliable agricultural technologies requires addressing two complementary questions: why does a treatment work, and does it remain effective under agricultural conditions? As summarized in Fig. 3, mechanistic evidence can range from associative observations, in which a cyanobacterial metabolite or trait coincides with a plant response without direct functional validation, to functional evidence based on defined compounds, dose–response relationships, and reproducible biological activity. Stronger causal evidence requires targeted genetic or biochemical perturbation linking a specific pathway or metabolite to the observed phenotype. These levels should not be interpreted as a strictly linear hierarchy, because mechanistic validation does not necessarily guarantee agronomic performance, just as field efficacy does not establish molecular causality.

Translational validation therefore represents a complementary axis, progressing from controlled laboratory experiments to greenhouse and field conditions (Fig. 3). Laboratory studies are valuable for establishing proof of concept and resolving physiological or mechanistic responses. In contrast, greenhouse experiments enable formulation testing and evaluation of plant performance under semi-controlled conditions, including interactions with soil and resident microbiota. Ultimately, field trials are required to determine agronomic performance, consistency across environments, and performance relative to conventional inputs. For example, *Nostoc carneum* and *N. commune* combined with 50% of the recommended fertilizer produced some rice yield responses comparable to full fertilization in a pot experiment (Chittapun et al. 2018), but such results do not establish equivalent field performance or product quality.

Importantly, biostimulant performance is context-dependent, reflecting interactions among cyanobacterial strain, host genotype, formulation, soil properties, and environmental conditions. In maize, genotype × inoculant × environment interactions significantly influenced available Zn, organic carbon, glomalin, available N, and polysaccharides, while cyanobacterial inoculation increased yield by 10–38% and improved soil-fertility indicators (Prasanna et al. 2021). Species-specific responses to cyanobacterial bio-priming were also observed in *Eucalyptus gamophylla*, *Grevillea wickhamii*, and *Triodia wiseana* during dryland restoration (Chua et al. 2020). Soil pH and other edaphic factors further shape cyanobacterial abundance and function (Kour et al. 2024), while locally isolated strains may exhibit greater tolerance to regional water limitation and osmotic stress (Nisha et al. 2018). Collectively, these observations emphasize that strain selection and validation should be performed within defined crop–soil–environment contexts rather than assuming broadly transferable efficacy.

This context dependence also extends to formulation and delivery strategy. Foliar spraying, seed soaking (priming), and seed coating with cyanobacterial or microalgal biomass-derived preparations can produce distinct physiological responses, with efficacy strongly influenced by dose and exposure time (Godlewska et al. 2019). Foliar applications primarily affect shoot growth, photosynthetic performance, and nutrient accumulation, whereas seed-based treatments such as soaking and coating can modulate early development, biomass production, and biofortification potential (Godlewska et al. 2019). These findings reinforce the need to optimize the strain–host–formulation–soil–environment combination rather than treating inoculant identity as the sole determinant of performance.

In parallel, terrestrial cyanobacteria originating from biological soil crusts have been shown to improve soil structure and functionality, a key objective in restoring degraded dryland ecosystems using indigenous soil cyanobacteria (Muñoz-Rojas et al. 2018; Jiménez-González et al. 2022). An outdoor study in degraded drylands demonstrated that combining cyanobacterial inoculation with plant-based amelioration strategies (*Macrochloa tenacissima* and *Plantago ovata*) significantly enhances biocrust establishment and soil restoration. These treatments improved cyanobacterial growth and soil properties, including aggregate stability and organic carbon content, compared to inoculation alone (Alameda-Martín et al. 2024). Recent work has underscored the effectiveness of indigenous strains as biofertilizers in agricultural areas, providing a foundation for future genetic, metabolic, or synthetic biology approaches for tailoring cyanobacterial functions to specific agroecosystems.

Safety assessment should proceed in parallel with mechanistic and translational validation rather than being considered only after agronomic efficacy has been established. As discussed in Sect.  4.3, candidate strains and formulations require strain-level assessment of cyanotoxin biosynthetic potential and, where appropriate, chemical confirmation, together with consideration of the regulatory framework applicable to the intended product and use. Integrating safety early in development is particularly important because cyanobacterial taxonomic identity alone does not establish an individual strain’s toxicological status.

Commercial translation remains comparatively limited. A dedicated market survey by Santini et al. (2021) identified three commercial formulations based on *Arthrospira* (or *Limnospira*) and several products containing unspecified cyanobacterial or microalgal biomass. However, publicly available information often does not disclose strain identity, formulation composition, or the specific bioactive components responsible for claimed effects, limiting classification based on mechanistic evidence. Commercialization is further constrained by production and processing costs, scalability, quality control, safety assessment, and regulatory requirements (Righini et al. 2022). These limitations highlight a persistent gap between experimentally identified cyanobacterial traits and standardized, mechanistically characterized agricultural products.

Therefore, broad agricultural deployment should not be viewed as the endpoint of a simple laboratory-to-field progression. Reliable cyanobacterial biostimulants require the convergence of mechanistic support, reproducible agronomic performance, appropriate formulation, and safety assessment within defined crop–soil–environment contexts (Fig. 3). Integrating low-cost cultivation and harvesting, standardized formulation and dosage, field validation, and techno-economic assessment will be essential for translating mechanistic knowledge into scalable cyanobacteria-based agricultural technologies (do Nascimento et al., 2019; Sun et al. 2026).

## Conclusions and future research priorities

Cyanobacterial diversity itself represents an underexploited resource. Despite growing evidence supporting the use of cyanobacteria in agricultural practices, several unresolved mechanistic questions continue to constrain the rational and predictable application of cyanobacteria as biostimulants. One central knowledge gap concerns the identity and specificity of cyanobacterial metabolites that act directly on plant tissues versus those that act indirectly by modulating the soil and rhizosphere microbiome. For many candidate metabolites, current evidence remains associative, with metabolite detection and plant responses reported concurrently but without functional or causal validation. Future studies should therefore move beyond co-occurrence by combining purified compounds or defined fractions with dose–response assays and, where feasible, genetic or targeted metabolic perturbation. In particular, the extent to which cyanobacterial compounds interact with plant hormonal networks, immune signaling pathways, and nutrient transport systems remains insufficiently understood. Future studies should determine whether specific plant receptors perceive candidate cyanobacterial elicitors and resolve downstream signaling events, including Ca²⁺ dynamics, MAPK activation, transcription-factor regulation, and crosstalk among SA-, JA-, auxin-, and ROS-dependent pathways. Disentangling these direct and microbiome-mediated effects of species- and strain-specific interactions is essential to move from empirical applications to mechanism-driven biostimulant design.

The context dependence of cyanobacterial effects, including soil properties, crop genotype, environmental conditions, and multitrophic interactions in the rhizosphere, poses a major challenge to reproducibility and scalability. Moreover, the long-term ecological consequences of repeated cyanobacterial applications, including potential shifts in microbial community structure and nutrient cycling dynamics, remain largely unexplored and demand future investigation. Addressing these challenges will require integrative approaches combining genomics, transcriptomics, proteomics, and metabolomics with advanced plant phenotyping and long-term, spatially and temporally replicated greenhouse and field experiments (Crandall et al. 2020; Doni et al. 2022; Gothe et al. 2024). Such studies should evaluate not only crop performance but also soil nutrient dynamics, microbiome responses, persistence of introduced cyanobacteria, and consistency across environmental conditions.

From an applied perspective, future research priorities should include: (i) isolation and functional characterization of taxonomically and metabolically diverse, locally adapted strains; (ii) standardized characterization of whole-cell, extract, metabolite-based, and immobilized formulations, including composition, stability, shelf life, and application dose; (iii) optimization of low-input cultivation, biomass harvesting, and formulation strategies suitable for scale-up; (iv) evaluation of nutrient release, biomass mineralization, and persistence under contrasting soils and climates; (v) multi-location and multi-season field validation to establish reproducibility across crop genotypes and management systems; and (vi) early integration of strain-level biosafety assessment, including cyanotoxin biosynthetic potential and chemical confirmation where relevant. These steps are essential for translating laboratory findings into reliable agronomic technologies (Bibi et al. 2024; Sun et al. 2026).

Looking ahead, predictive frameworks integrating cyanobacterial metabolomic signatures, plant physiological responses, soil properties, and microbiome indicators could guide the selection of strains and formulations for specific crop–environment combinations. Synthetic biology and microbial engineering may further enable optimization of validated traits or the construction of consortia with complementary functions, but development must proceed alongside ecological, biosafety, and regulatory assessment (Bharti and Suryavanshi 2021). Ultimately, progress will depend less on expanding the catalog of reported cyanobacterial benefits than on establishing which strains, metabolites, and mechanisms produce reproducible effects, under which environmental conditions, and with an acceptable safety profile. This transition from descriptive potential to mechanistically validated and field-reproducible performance will be critical for the responsible development of cyanobacteria-based agricultural technologies.

## Data Availability

No datasets were generated or analysed during the current study.
